# The Abundance and Distribution of the *acdS* Gene in Microbial Communities from the Rhizosphere of *Copiapoa solaris*, a Native Cactus in the Arid Coastal Region of Antofagasta, Chile

**DOI:** 10.3390/microorganisms13071547

**Published:** 2025-07-01

**Authors:** Mayra Cayo, Francisco Solís-Cornejo, Andrés Santos, Pedro Zamorano, Bernardita Valenzuela

**Affiliations:** 1 Programa de Magíster en Ecología de Sistemas Acuáticos, Facultad de Ciencias del Mar y Recursos Biológicos, Universidad de Antofagasta, Antofagasta 1240000, Chile; mayra.cayo@uamail.cl; 2 Programa de Doctorado en Ciencias Aplicadas Mención Sistemas Acuáticos, Facultad de Ciencias del Mar y Recursos Biológicos, Universidad de Antofagasta, Antofagasta 1240000, Chile; 3 Laboratorio de Microorganismos Extremófilos, Instituto Antofagasta, Universidad de Antofagasta, Angamos 601, Casilla 170, Antofagasta 1240000, Chile; francisco.solis@uamail.cl (F.S.-C.); pedro.zamorano@uamail.cl (P.Z.); 4 Departamento Biomédico, Facultad de Ciencias de la Salud, Universidad de Antofagasta, Antofagasta 1240000, Chile; 5 Departamento Ciencias Básicas, Facultad de Medicina, Universidad de la Frontera, Temuco 4780000, Chile; andres.santos@ufrontera.cl; 6 Laboratorio de Bioinformática y Microbiología Aplicada, Centro de Excelencia en Medicina Traslacional, Universidad de la Frontera, Temuco 4780000, Chile; 7 Departamento de Educación, Facultad de Educación, Universidad de Antofagasta, Antofagasta 1240000, Chile

**Keywords:** *acdS* gene, *Copiapoa solaris*, rhizosphere

## Abstract

*Copiapoa solaris* is an endemic cactus species from the Antofagasta region, Chile, thriving in arid coastal ecosystems known as “fog oases,” where the rising marine moisture is the primary water source. This study investigates the role of microbial communities associated with the rhizosphere of *C. solaris* in adapting to extreme environmental conditions, particularly focusing on the *acdS* gene, which encodes ACC deaminase—an enzyme that reduces ethylene production under stress. This research aims to elucidate the gene’s contribution to the adaptation of *C. solaris* in these challenging environments. Samples were collected from three sites (El Cobre, Quebrada Botija, and Quebrada Izcuña) that differ in relative humidity, temperature, and topography. Environmental DNA was extracted, phylogenetic diversity was analyzed, and metagenomic annotation of the *acdS* gene was conducted. The *acdS* gene was detected in all samples, with the highest relative abundance at Quebrada Izcuña (0.05%), characterized by low relative humidity (<70%) and severe water stress. Phylogenetic analysis revealed conserved sequences across sites, while taxonomic and alpha diversity were similar among them. However, beta diversity indicated that Quebrada Izcuña was the least homogeneous, hosting distinct taxa potentially associated with stress mitigation. The *acdS* gene was detected on plasmids at El Cobre and Quebrada Izcuña, suggesting its potential mobility within the metagenome. The results of this study highlight the intricate relationships between microbial communities and the resilient cactus species *C. solaris* in extreme environments. The conservation and abundance of the *acdS* gene, particularly in low-humidity conditions, suggest its vital role in facilitating stress tolerance through microbial interactions. Understanding these dynamics is crucial for developing strategies to enhance plant resilience in arid ecosystems, with potential applications in sustainable agriculture and ecosystem management under changing climatic conditions.

## 1. Introduction

Aquatic systems, including oceans and lakes, are vital for the hydrological cycle but are scarce in arid environments, where annual precipitation often falls below 500 mm [1,2]. In these regions, “fog oases” serve as essential sources of moisture, capturing atmospheric water to help mitigate water scarcity [3,4].

The genus *Copiapoa* is endemic to Chile and distributed from the northern coastal deserts to the central regions of the country. *C. solaris* thrives in small patches of the coastal mountain range of the Antofagasta region, moisturized by these fog oases, which are uniquely adapted to the hyper-arid coastal environment. Due to the limited geographical distribution, this species is notable for its ecological importance and vulnerability to habitat loss caused by climate change and anthropogenic activities, including mining and road construction [5]. Cacti have a symbiotic relationship with plant growth-promoting rhizobacteria (PGPR), which enhance resilience to abiotic stressors like drought and salinity [6]. PGPR can produce phytohormones, solubilize phosphates, and synthesize siderophores, among other benefits [7].

In general, plants produce 1-aminocyclopropane-1-carboxylic acid (ACC) as part of their ethylene production cycle, a hormone involved in fruit ripening, leaf senescence, and stress response. This includes abiotic stress factors like drought, salinity, and extreme temperatures. Although ethylene serves important functions, its excessive accumulation during stress can lead to senescence or cellular damage [8,9,10].

One key mechanism by which plant growth-promoting rhizobacteria (PGPR) support plants under stress is through the activity of ACC deaminase, an enzyme encoded by the *acdS* gene. ACC deaminase lowers ethylene levels in plants, alleviating stress and promoting growth [11,12]. The *acdS* gene is widely distributed among bacterial taxa and is often associated with mobile genetic elements, such as plasmids, facilitating horizontal transfer [13,14]. Its presence in rhizosphere bacterial communities associated with various plants, including cacti, has been extensively documented [15].

The abundance and distribution of the *acdS* gene in microbial communities are influenced by environmental factors such as water availability, salinity, and soil composition [16,17]. Metagenomic analyses have highlighted the diversity of ACC deaminase-producing bacteria and their critical role in enhancing plant resilience in challenging environments [15,18]. However, the distribution and diversity of the *acdS* gene in hyper-arid regions, particularly in the rhizosphere of *C. solaris*, remain poorly understood. Investigating these microbial communities is essential for developing strategies to mitigate environmental stress and conserve these ecosystems.

Research indicates that bacterial communities with high ACC deaminase activity promote the survival and growth of host plants under extreme conditions [19,20]. It is hypothesized that lower water availability in fog oases will correlate with a higher abundance of the *acdS* gene in the rhizosphere of *C. solaris*. This hypothesis is supported by studies on the gene’s adaptive role in extreme environments [17] and the importance of plant–microbe interactions in arid conditions [6,12]. Understanding these microbial dynamics is vital for uncovering the mechanisms that support plant survival and resilience in harsh environments.

The objective of this study was to analyze the phylogenetic diversity and abundance of the *acdS* gene in bacterial communities associated with the rhizosphere of *C. solaris* across different distribution zones. To achieve this, we mapped the distribution of *C. solaris* in relation to environmental factors such as relative humidity, temperature, altitude, and solar exposure. Additionally, we characterized the taxonomic structure and *acdS* gene diversity and abundance in these microbial communities using metagenomic (shotgun) analysis. This study further aimed to correlate these microbial traits with environmental parameters, providing insights into the role of microbial adaptations in arid ecosystems. This research highlights the importance of understanding plant–microbe interactions and their contribution to plant survival in extreme conditions, advancing knowledge of adaptive mechanisms in fog oasis ecosystems.

## 2. Materials and Methods

### 2.1. Sampling Sites

**Figure 1:**
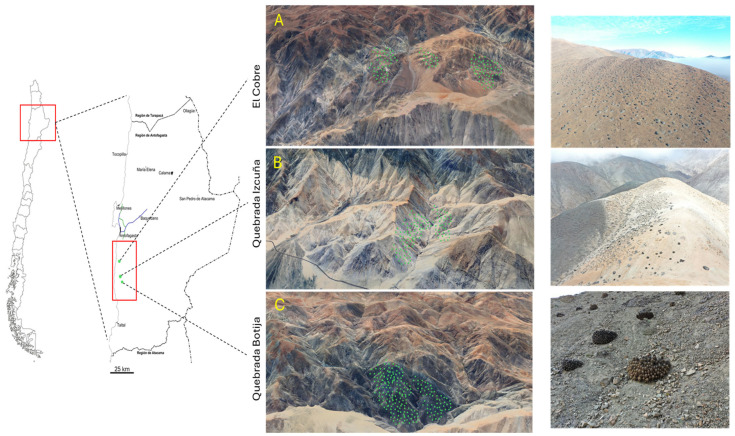
Geographic distribution of *Copiapoa solaris* in three sampling sites located in the Antofagasta region, Taltal Commune. The 25 km study area is delimited in red, with the sites as follows. (**A**) El Cobre; (**B**) Quebrada Izcuña; (**C**) Quebrada Botija.

This study was conducted in three areas located in the Antofagasta region, El Cobre (24°29′ S, 70°49′ W), Quebrada Izcuña (24°42′ S, 70°52′ W), and Quebrada Botija (24°50′ S, 70°53′ W), all situated near Caleta El Cobre (24°15′ S, 70°31′ W), as shown in Figure 1. These sites are significantly influenced by marine fog, locally known as “Camanchaca” [21]. In each area, plant species were identified, and soil samples from the rhizosphere were collected. Three samples, each weighing approximately 20 g, were taken at a 5 cm distance from the roots, with a 10 m interval between each sample [22].

### 2.2. Environments Parameters

Temperature and relative humidity were measured at the studied sites using a Hobo U23-002A Pro V2 data logger sensor (Onset Computer Corporation, Bourne, MA, USA), with measurements every 2 h over a three-month period. Additionally, rhizosphere humidity was measured in situ using a VB brand garden humidity probe. For UV data, the online platform of the Meteorological Directorate of Chile was accessed, mainly from the Taltal Station, for data extraction. For the distribution of *Copiapoa solaris*, a Drone (Da-jiang innovations science and technology (DJI), Model: RC1B, Shenzhen, China) was used, which flew over the sites where the presence of *C. solaris* has been described, carrying out a complete survey for each study site. The images obtained were then analyzed to generate a correct distribution map of the species.

### 2.3. Environmental DNA Extraction and Shotgun Sequencing

To characterize the bacterial community associated with the rhizosphere of *C. solaris*, total DNA was extracted from 0.25 g of soil using the “DNeasy PowerSoil Pro-Kit” (QIAGEN, Hilden, Germany), following the manufacturer’s protocol. The concentration of DNA in each sample was quantified using the Qubit dsDNA HS Assay Kit (Invitrogen, Thermo Fisher Scientific, Waltham, MA, USA), with measurements taken using a Qubit 4 fluorometer (Thermo Fisher Scientific, Waltham, MA, USA). The samples were then sequenced using the Illumina NovaSeq 6000 platform. A TruSeq Nano DNA Library (350 bp) was prepared for sequencing. Library preparation and sequencing were conducted by Macrogen (Seoul, Republic of Korea).

### 2.4. Assembly and Taxonomy

The sequences obtained from the sequencing were previously filtered using Fastp [23], which were subsequently assembled using the Megahit V1.2.9 assembler [24]. To evaluate the quality of the assembly, the Quast v5.2.0 software [25] was employed, providing metrics such as assembly quality, the number of contigs per sample, and N50-L50 values, among others.

Taxonomic profiling was carried out using Kraken2 v1.1.1 [26], using the database MinusB, with the analysis conducted on the reads for each sample. The classification percentage for each sample and both the overall taxonomic profiles and the profiles of microorganisms that showed significant differences by sampling site were determined at the phylum, family, and genus levels. Furthermore, both the alpha and beta diversity of the samples were evaluated, and single-factor statistical comparisons using edgeR as a statistical method were conducted by sampling site to identify microbial genera that exhibited significant variation across sites.

### 2.5. acdS Gene Annotation

To identify the *acdS* gene, a database was initially constructed as a custom database, comprising over 1000 sequences from various microorganisms in which the *acdS* gene had been previously characterized. The Prodigal v2.6.3 software [27] was then employed to process the assembled metagenomic data for each sample, extracting the coding sequences (CDSs) for each. These CDSs were annotated using Diamond v2.1.9 software [28,29], comparing the obtained CDSs from each sample with the custom database. An identity cutoff of 60% and a minimum length of 300 amino acids were used to filter annotations and conduct further analyses. Finally, we calculated the relative abundance of the acdS gene as the total number of sequences per sample divided by the total number of genes predicted by Prodigal, as described by Van Goethem et al., 2018 [30].

### 2.6. Phylogenetic Analysis

After obtaining the amino acid sequences of the *acdS* gene, redundant sequences were grouped considering a 97% identity percentage using the cd-HIT v4.8.1 software, resulting in the selection of the most representative sequences for phylogenetic analysis. These sequences were aligned using MAFFT v7 [31], and a phylogenetic tree was subsequently constructed using MEGA 11 [32], focusing on the most conserved regions. Taxonomic information for each sample was considered, allowing for the identification of clustering patterns specific to each study site.

### 2.7. Mobile Element Reconstruction

The association of the *acdS* gene with mobile genetic elements was assessed. For this, mobile elements of each rhizosphere metagenome of *C. solaris* were reconstructed to identify the *acdS* gene. This was accomplished using the PLASMe v1.1 software [33], which utilizes assembled metagenomes for plasmid reconstruction. The generated files were filtered, with sequences longer than 800 nucleotides being considered for further analysis. To identify the *acdS* gene, Prodigal and Diamond software were employed, referencing the previously described database.

### 2.8. Principal Component Analysis (PCA) Correlation

For the correct correlation of the biological data obtained by metagenome sequencing such as the environmental data obtained (temperature, humidity), a principal component analysis (PCA) was performed to obtain a graph that showed the principal components, which are those that retain the most information delivered [34].

## 3. Results

### 3.1. Description Sampling Sites

**Figure 2:**
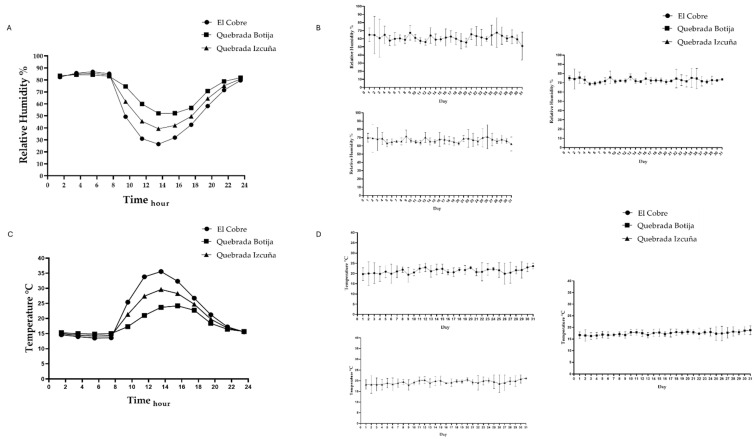
Environmental graphs of temperature (°C) and relative humidity (%) variation in the three study sites: El Cobre, Quebrada Botija, and Quebrada Izcuña. (**A**) The variation in relative humidity throughout the day is observed. It is observed that humidity is higher in the early morning and at night, reaching values above 80%, while at midday and during the afternoon, it decreases significantly. Quebrada Botija presents the highest humidity values compared to the other two sites. (**B**) The variation in relative humidity during a month at the three study sites. High daily fluctuation is observed at the three sites, with Quebrada Botija maintaining higher humidity levels compared to El Cobre and Quebrada Izcuña, which present lower and fluctuating values. In (**C**) the variation in temperature throughout the day is observed at the three sampling sites. A progressive increase in temperature is observed until a maximum is reached between 12:00 and 14:00 h. El Cobre presents the highest temperatures, followed by Quebrada Izcuña and Quebrada Botija, which show the lowest values. In (**D**) the daily variation in temperature is observed during a month at the three study sites. Constant fluctuation in temperature is observed, with El Cobre recording the highest values compared to the other sites, while Quebrada Botija maintains more stable and slightly lower temperatures.

The study sites are distributed along a 25 km transect between El Cobre and Quebrada Botija. Three rhizosphere samples were collected at each site, considering the vegetative state of *Copiapoa solaris* (alive, stressed, and dead), with each point described in Table 1. Additionally, a species distribution map was created based on the analyzed sites, as shown in Figure 1. To complement this study, environmental variables such as temperature and humidity were evaluated (Figure 2), revealing an inverse correlation. The average temperature ranged from a maximum of up to 30 °C to a minimum of 15 °C, while relative humidity showed maximum values exceeding 80% and minimum values below 50%, reflecting the environmental variability in the area.

### 3.2. Environmental DNA Extraction and Shotgun Sequencing

The sequenced samples generated more than 5 GB of data. These data were used for the assembly and taxonomic identification of each DNA sample obtained from the study sites. Taxonomic assignment was subsequently performed, allowing for the identification of diverse bacterial communities present in the rhizosphere of *C. solaris*. Sequencing results revealed that for all metagenome samples, between 38,628,228 and 49,070,206 reads were obtained as a result of sequencing, which translated to 5.8 and 7.4 GB of information.

### 3.3. Assemblies and Taxonomy

**Figure 3:**
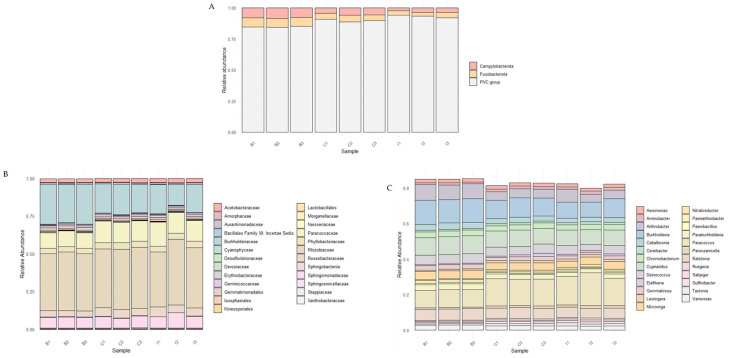
Taxonomic composition of the microbial community showing significant differences among the three study sites: Quebrada Botija (B1, B2, B3), El Cobre (C1, C2, C3), and Quebrada Izcuña (I1, I2, I3). (**A**) Relative abundance at the phylum level, where three predominant taxa were identified, *Campylobacterota*, *Fusobacteriota*, and *PVC group*, consistently present across all samples. (**B**) Relative abundance of the top 25 most abundant families, revealing a heterogeneous distribution, with *Rhizobiaceae* emerging as one of the dominant families across sites. (**C**) Relative abundance of the top 25 most abundant genera, also showing a heterogeneous distribution, with genera such as *Paraburkholderia*, *Paenibacillus*, and *Burkholderia* identified among the samples.

**Figure 4:**
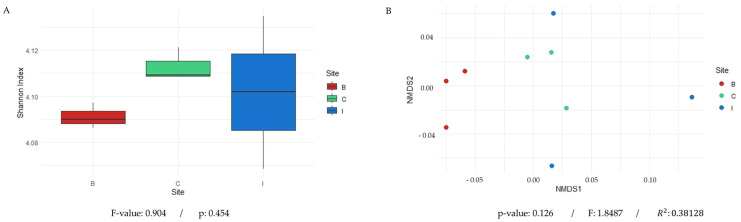
Analysis of microbial diversity across the three study sites: Quebrada Botija (B), El Cobre (C), and Quebrada Izcuña (I). (**A**) A representation of the alpha diversity of microbial communities at each sampling site, measured using the Shannon index, which showed similar values across sites, with greater variability among replicates at site I. This indicates that the statistical results yielded an **F-value of 0.904** and ***p* = 0.454**, suggesting no statistically significant differences in microbial diversity or in the abundance of the *acdS* gene among sites. (**B**) Non-metric multidimensional scaling (NMDS) analysis based on Bray–Curtis dissimilarity revealed partial clustering of samples according to their site of origin, highlighting differences in microbial composition. Samples from Quebrada Botija cluster in a distinct area of the plot, suggesting a unique microbial community compared to the other sites. In contrast, samples from El Cobre and Quebrada Izcuña exhibit greater dispersion, indicating higher variability in their microbial composition. The PERMANOVA analysis (999 permutations) showed that **38.1% of the variation** in microbial community structure is explained by sampling site (**R^2^ = 0.381**), although these differences were **not statistically significant** (***p* = 0.126**).

For the analysis of the samples, sequencing data were processed using the Fastp tool for quality filtering, followed by sequence assembly with Megahit. The results, presented in Table 2, detail the number of contigs generated (ranging from 60,000 to 119,000) for each metagenome, as well as the N50 and L50 values, which serve as key indicators for assessing assembly quality. In parallel, the taxonomic profiling of the rhizosphere samples of *C. solaris* was carried out, revealing that only between 6% and 8% of the total sequences could be classified. At the phylum level, as shown in Figure 3, microorganisms showing statistically significant differences between samples were identified, with a predominance of the *PVC group*, *Fusobacteriota*, and *Campylobacteriota* across the three study sites. At the family level, the relative abundance of the 25 most abundant families was observed, showing a heterogeneous distribution among the samples, with *Rhizobiaceae* and *Bacillales*, among others, emerging as the most dominant across the different sites. At the genus level, a strong predominance of certain microorganisms was found, with *Paraburkholderia*, *Paenibacillus*, and *Burkholderia*, among others, standing out as prevalent genera in the analyzed samples. In parallel, a taxonomic analysis considering all present taxa (including those that did not show differences between sampling sites) was performed, in which at the phylum level, as shown in Figure S1, the predominance of *Pseudomonadata* and *Terrabacteria* was identified across the three study sites. Additionally, the analysis of low-abundance phyla (less than 1%) revealed that the *PVC group* was the most representative within this fraction. Taxonomic analysis at the family level showed a relatively homogeneous distribution among samples, with a notable abundance of families such as *Rhizobiaceae*, *Mycobacteriales*, *Streptosporangiales*, and *Burkholderiaceae*. At the genus level, a higher prevalence of *Nonomuraea*, *Rhizobium*, *Streptomyces*, *Azospirillum*, *Tsukamurella*, *Shinella*, *Paracoccus*, *Rhodococcus*, *Burkholderia*, *Nocardia*, *Sinorhizobium*, *Cupriavidus*, and *Methylobacterium* was observed. It is worth noting that these genera identified in all samples have been previously described as plant growth-promoting bacteria in various plants and crops. Regarding microbial diversity, Figure 4A,B present the alpha diversity of the microbial communities using the Shannon index for the three sampling sites (B: Quebrada Botija; C: El Cobre; I: Quebrada Izcuña). Slight variation was observed among the sites, with the lowest value recorded at site B and the highest values at sites C and I, suggesting greater microbial diversity at the latter. However, when a statistical analysis was performed, the Shannon diversity index showed an F-value of 0.904, indicating a low difference among the compared sites, and a *p*-value of 0.454, suggesting that there was no statistically significant difference among the study sites. On the other hand, Figure 4C shows the non-metric multidimensional scaling (NMDS) analysis based on Bray–Curtis distance, where partial clustering of the samples according to their site of origin is evident. This reveals differences in the microbial community structure, with El Cobre and Quebrada Izcuña showing greater dispersion, indicating higher variability in microbial composition. In addition, a PERMANOVA analysis was conducted to confirm and support the differences found in beta diversity. Table S1 shows the results of the analysis with 999 permutations, indicating that the samples presented a 38.1% variation in microbial community structure (R^2^ = 0.381). It is worth noting that although differences exist, they are not statistically significant (*p* = 0.126). These findings suggest that the environmental conditions of each site may influence the diversity and composition of soil microbiota, reinforcing the importance of factors such as moisture and nutrient availability in shaping rhizosphere microbial communities.

### 3.4. acdS Gene Annotation

Analysis of *acdS* gene abundance in rhizosphere samples showed that both the annotation and abundance of this gene remained below 1% at all sites evaluated. Specifically, *acdS* gene abundance was 0.0184% at Quebrada Botija, 0.0314% at El Cobre, and 0.0474% at Quebrada Izcuña. This distribution pattern indicates an inverse correlation between ambient relative humidity and gene abundance. Quebrada Botija, characterized by high relative humidity, had the lowest *acdS* gene abundance, while El Cobre and Quebrada Izcuña—sites with low humidity conditions—had higher *acdS* gene abundance. To assess whether these differences were statistically significant between sites, an analysis of variance (ANOVA) was performed. The results indicated no significant differences in the abundance of the *acdS* gene among the sites evaluated (F = 0.904; *p* = 0.454). This suggests that, although an apparent trend in the gene’s distribution associated with relative humidity was observed, the variations were not statistically significant within the analyzed dataset. However, these findings suggest that in hyper-arid environments, stressors such as drought and water scarcity may be closely associated with the presence of the *acdS* gene, potentially indicating an adaptive role in the response of microbial communities to adverse environmental conditions.

### 3.5. Phylogenetic Analysis

**Figure 5:**
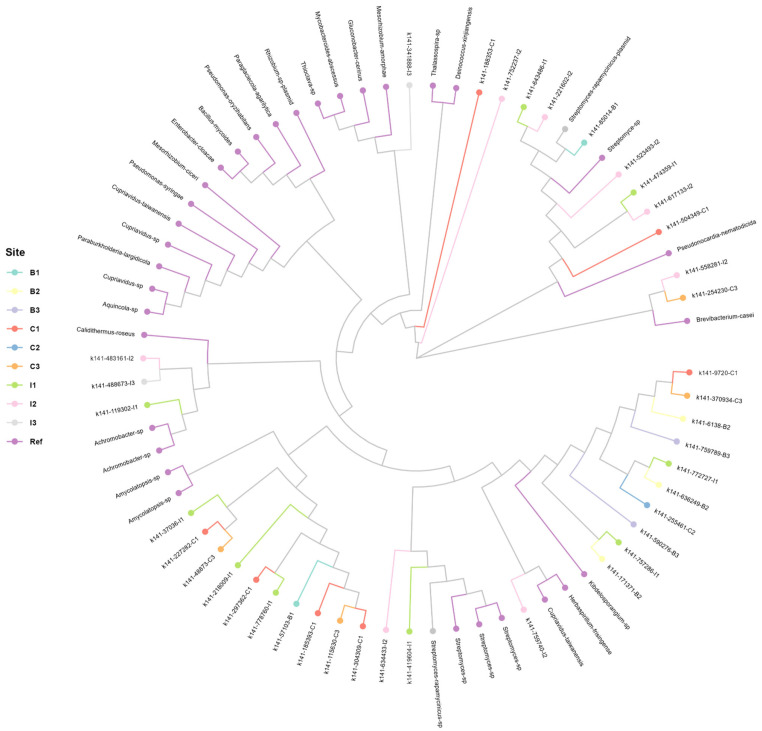
Phylogenetic analysis of taxonomic diversity in the sequences obtained from the *acdS* gene with the presence of different bacterial genera such as *Cupriavidus*, *Aquincola*, *Achromobacter*, *Amycolatopsis*, and others. There is no clear exclusivity by sampling site, suggesting that some taxa are shared between the different study sites.

The analysis of the *acdS* gene is shown in Figure 5, which presents the generated phylogenetic tree, revealing the significant taxonomic diversity of the *acdS* gene at the genus level, thus identifying the presence of the gene in a particular study site or in a specific genus of microorganisms that inhabit the rhizosphere of this cactus. However, all the samples analyzed belong to the same family, suggesting that the presence and conservation of the *acdS* gene could be related to the function of the microorganisms present in the rhizosphere, since the microorganism–cactus interaction is crucial for the survival of the species in periods of stress such as water scarcity. The analysis of the generated clusters indicates that there is no exclusivity in the presence of the *acdS* gene per sampling site, which means that the presence of the *acdS* gene is shared between the different study sites (B1-B3, C1-C3, I1-I3). This homogeneous distribution suggests that the gene’s function could be associated with common environmental factors across sites rather than ecosystem-specific characteristics. Furthermore, taxa of interest such as *Cupriavidus*, *Aquincola*, *Brevibacterium*, *Achromobacter*, and *Amycolatopsis* were identified, showing a dispersed distribution in the phylogenetic tree. This indicates that the presence of the *acdS* gene in the rhizosphere is not limited to a specific lineage but can be found in various bacterial genera with common adaptive functions. The phylogenetic structure of the *acdS* gene in the analyzed rhizosphere reveals a shared distribution across the study sites, with taxonomic diversity at the genus level but conservation at the family level. These results suggest that the presence of the gene could be influenced by general environmental factors, allowing its conservation in multiple microbial groups present in the rhizosphere.

### 3.6. Mobile Element Reconstruction

For the identification of the *acdS* gene in mobile elements of the *C. solaris* rhizosphere, a plasmid reconstruction analysis was conducted. Table 3 presents the number of plasmids identified in each sample. Variability in the number of recovered plasmids was observed across the different sampling sites, with sample I2 showing the highest number of plasmids (6.121 plasmids), while sample C3 had the lowest (2.502 plasmids). To identify the *acdS* gene, Prodigal v2.6.3 and Diamond v2.1.9 software were used, referencing the previously described database. The results, detailed in Table 4, confirm the presence of the *acdS* gene in plasmids from different samples, with identity levels ranging from 60% to 76%. Notably, sample I1 contained two plasmids carrying the *acdS* gene, with coverage of 204 and 316 amino acids, respectively.

These findings indicate that the *acdS* gene is associated with mobile genetic elements in the *C. solaris* rhizosphere, suggesting its possible horizontal transfer among soil microorganisms. The high number of plasmids identified in certain samples, such as I2, suggests that the propagation of the *acdS* gene could be influenced by the abundance and diversity of plasmids present in the environment. This further indicates that the *acdS* gene spreads through genetic transfer mechanisms, potentially playing a key role in the adaptation of microbial communities to specific environmental conditions in the *C. solaris* rhizosphere.

### 3.7. Principal Component Analysis (PCA) Correlation

**Figure 6:**
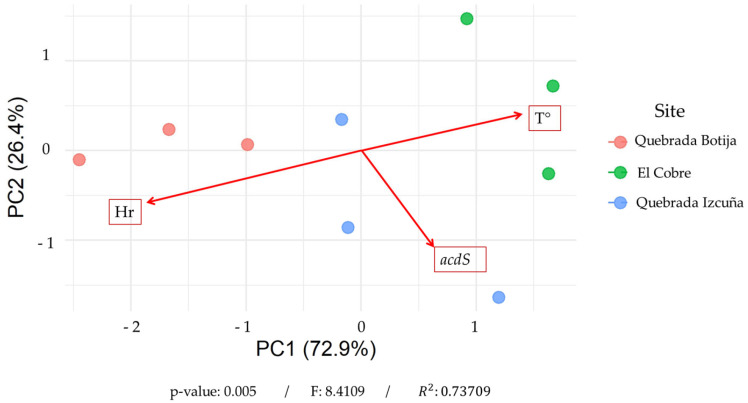
Principal component analysis (PCA) revealed that the first two components explain 99.38% of the total variability, with PC1 accounting for 72.93% and PC2 for 26.45%. This high proportion indicates that the environmental variation can be robustly represented in a two-dimensional space. The samples show a clear separation in the multivariate space according to locality, suggesting the existence of distinct environmental conditions among the sampled sites. This pattern was statistically confirmed by a PERMANOVA analysis, which demonstrated significant differences between localities (F = 8.4109; R^2^ = 0.73709; *p* = 0.005). The model explained 73.7% of the total variability in the environmental variables, indicating that geographic locality acts as a primary determinant of environmental variation in this system.

To correlate the biological data obtained through metagenomic sequencing with the environmental data collected (temperature and relative humidity), a principal component analysis (pCa) was performed. Figure 6 shows the distribution of the samples based on the first two principal components (PC1 and PC2), which together explain 99.38% of the total variability—PC1 accounting for 72.93% and PC2 for 26.45%. This high proportion reflects the influence of environmental variation. The clear separation of samples by sampling site is observed. Samples from Quebrada Izcuña cluster in the lower right quadrant of the plot, those from El Cobre in the upper right, and those from Quebrada Botija on the left side. The vectors representing humidity (Hr) and temperature (T°) indicate the influence of these environmental variables on sample distribution. Quebrada Botija, located at the end of the humidity vector, is associated with more humid environments, while Quebrada Izcuña and El Cobre show a stronger correlation with temperature. Notably, an inverse relationship is observed between relative humidity and the presence of the *acdS* gene, with the higher abundance of this gene in Quebrada Izcuña and El Cobre—sites characterized by lower humidity and higher temperatures. In contrast, Quebrada Botija, the most humid site, shows a weaker association with the *acdS* gene. Additionally, a PERMANOVA analysis was conducted (Table S2), revealing significant differences among the sampling sites (F = 8.41; R^2^ = 0.737; *p* = 0.005). The model explained 73.3% of the total variability in the environmental variables. These findings suggest that the distribution of the *acdS* gene in the rhizosphere of *C. solaris* is influenced by environmental factors, with higher abundance in more arid and warmer locations such as Quebrada Izcuña and El Cobre. This indicates that the geography of the study area acts as a key determinant of variation in this novel ecosystem and may point to the potential adaptive role of the *acdS* gene in response to water and thermal stress conditions.

## 4. Discussion

### 4.1. Environmental Influence on Microbial Diversity in the Rhizosphere of Copiapoa solaris

The microbial communities associated with the rhizosphere of *Copiapoa solaris* exhibit a significant relationship with environmental factors—such as temperature and relative humidity—present in the arid coastal zone of the Antofagasta region and the microbial composition within the rhizosphere of this species [35,36]. The variability in environmental conditions (temperature and relative humidity) has been proven to be a key variable influencing microbial community structure across the three sampling sites. Notably, an inverse correlation has been identified between relative humidity and the abundance of the *acdS* gene, indicating that more arid conditions favor the presence and expression of this gene in rhizosphere-associated microorganisms, likely due to its role in mitigating water stress [37,38]. These findings are consistent with previous studies conducted in arid ecosystems, where the presence of drought-adapted bacteria is linked to the production of ACC deaminase, a key enzyme in the environmental stress response in plants [16,20,39].

The presence of this gene in the rhizosphere of *C. solaris* suggests that microorganisms associated with this species may play a crucial role in its adaptation to the region’s extreme climatic conditions. Previous studies have shown that plant growth-promoting bacteria enhance plant resistance to water stress [40,41,42], thereby providing a competitive advantage in arid environments [43,44]. Moreover, microbial diversity in the rhizosphere [45,46] can influence the plant’s ability to access essential nutrients, facilitating its survival in soils with low fertility [47,48,49]. This analysis also suggests that the microbial community of *C. solaris* has developed specific adaptive mechanisms, which could explain its distribution in extremely dry habitats.

### 4.2. Taxonomic Composition and Functional Implications

The taxonomic profiling revealed that microbial communities showing significant differences among the study sites of *C. solaris* were dominated by the phyla *PVC group* [50], *Fusobacteriota* [51], and *Campylobacterota* [52]. These phyla have been widely reported as both endophytic and rhizosphere-associated bacteria. Additionally the overall microbial communities present in the rhizosphere of *C. solaris* were largely dominated by the phyla *Pseudomonadota* [53] and *Terrabacteria* [54], with additional representation of *Actinobacteria* [55], *Betaproteobacteria* [56], and *Bacteroidetes* [57,58]. These phyla have been widely reported in arid and hyper-arid environments [59,60,61] and are known to play key roles in promoting plant growth and stress tolerance to various stress factors such as drought [15,62,63]. The ability of these microorganisms to improve water uptake and nutrient absorption is a critical trait in ecosystems where resource availability is limited [64,65]. The microbial composition of the rhizosphere can influence numerous processes, including the modulation of the plant immune system [66,67] and protection against soil pathogens [68,69,70].

Moreover, the identification of bacterial genera such as *Rhizobium* [71], *Burkholderia* [72], *Paenibacillus* [73,74], and *Streptomyces* [75], known for their role in nitrogen fixation [76,77] and phytohormone production [78,79], suggests that these bacteria may contribute to the survival of *C. solaris* in its natural habitat. Their ability to modulate plant growth and enhance resilience under adverse conditions highlights the ecological importance of plant–microbe interactions in arid environments [6]. The metagenomic evidence presented in this study demonstrates that microbial communities in arid soils exhibit specific adaptive traits that enable them to survive under extreme stress conditions, playing a fundamental role in the ecology of xerophytic plants [80,81].

### 4.3. Distribution and Mobility of the acdS Gene

The *acdS* gene was detected at all study sites, albeit with an abundance of less than 1%. However, its presence was more pronounced in Quebrada Izcuña and El Cobre, suggesting that its presence is influenced by low water availability and other environmental stress factors [82]. The relationship between the abundance of this gene and relative humidity is consistent with previous findings indicating that *acdS*-harboring bacteria play a key role in adaptation to water stress [83,84], promoting plant growth under drought conditions. The ecological diversity of these bacteria is an important aspect to consider, as their colonization ability in different habitats may be determined by their interaction with soil microbiota and local environmental conditions [85].

Phylogenetic analysis of the *acdS* gene indicated that it is conserved within specific bacterial families [16], but it is widely distributed among different genera, including *Cupriavidus* [86], *Aquincola* [87] and *Achromobacter* [88,89]. These genera have been reported as endophytic bacteria [78,90] capable of acting as plant growth-promoting bacteria (PGPB), as they are involved in regulating the overproduction of ethylene caused by stress. Endophytic bacteria, like those present in the rhizosphere and described as PGPR (plant growth-promoting rhizobacteria), show great potential for the bioremediation of environments contaminated with heavy metals or facing environmental stress [91,92]. This distribution suggests that the *acdS* gene is not exclusive to a particular lineage but is associated with a key function in microbial adaptation to arid environments [14]. Previous studies have reported similar patterns of stress gene conservation in microbial communities from soils with high climatic variability [11]. The identification of multiple bacterial genera harboring this gene also reinforces the hypothesis that adaptation to water stress is a trait shared by diverse species in arid environments [93,94].

### 4.4. Horizontal Gene Transfer and Metagenomic Assembly

The identification of the *acdS* gene in plasmids suggests its potential for horizontal gene transfer (HGT), a mechanism that facilitates the dissemination of adaptive traits within the microbial community. Notably, in sample I1, two plasmids carrying the *acdS* gene were identified, indicating that genetic elements associated with stress adaptation can mobilize within the microbial community [95,96,97] and play a crucial role in microbial adaptation to environmental stress [98,99].

Previous studies have demonstrated that genes involved in drought tolerance, including *acdS* [100,101,102], are frequently linked to mobile genetic elements, allowing them to spread across different ecological niches and diverse bacterial species [13]. The presence of these plasmids in samples with lower humidity suggests that horizontal gene transfer may be favored in more arid environments, where selective pressure is higher and competition for scarce resources drives genetic diversification.

### 4.5. Principal Component Analysis and Environmental Correlation

The principal component analysis (PCA) confirmed the relationship between environmental variables and the composition of microbial communities in the rhizosphere of *Copiapoa solaris*. The clear separation of samples according to their sampling sites indicates that local geography acts as a key ecological determinant in shaping soil microbiota. The high proportion of variance explained by the first two principal components (PC1 = 72.93%; PC2 = 26.45%) supports this observation, suggesting that a substantial portion of the observed biological variation can be attributed to specific environmental conditions. The PERMANOVA analysis revealed significant differences between the sampling sites (F = 8.41; R^2^ = 0.737; *p* = 0.005), accounting for 73.3% of the total variability in the environmental variables. This finding reinforces the influence of geography and environmental gradients on the functional structure of microbial communities, suggesting that the observed patterns are not random but rather the result of biological responses to defined environmental pressures. Notably, samples from Quebrada Botija, characterized by higher humidity levels, exhibited a distinct community structure compared to those from El Cobre and Quebrada Izcuña, which were associated with higher temperatures and lower humidity. The strong correlation between temperature and the abundance of the ***acdS*** gene further supports the idea that microbial communities in these sites have developed specific strategies to cope with thermal and hydric stress [38,95,103]. These findings are consistent with studies conducted in other arid regions around the world, where environmental pressure has been shown to shape microbial diversity and promote the selection of genes involved in stress resistance [17,18].

### 4.6. Implications for Conservation and Biotechnological Applications

The results obtained in this study have important implications for the conservation of plant species in arid environments [104,105,106]. The presence of *acdS* in specific microbial taxa highlights its role in promoting plant drought resistance [107]. Furthermore, the identification of rhizosphere-associated microorganisms with potential benefits for *C. solaris* enables the conservation of this endangered species [108,109] and opens new opportunities for the development of biofertilizers based on microbial consortia adapted to extreme aridity conditions.

Agricultural biotechnology can benefit from the knowledge generated in this study, particularly in the development of microbial inoculants designed to enhance crop resilience in water-scarce regions [110,111]. In this regard, the exploitation of *acdS*-harboring bacteria could represent a viable strategy to increase drought tolerance in agriculturally and ecologically important plant species [11,19].

## 5. Conclusions

This study highlights the fundamental role of microbial communities in the rhizosphere of *C. solaris* and their adaptation to extreme environmental conditions. The detection of the *acdS* gene in bacterial taxa associated with stress resistance suggests that this gene plays a key role in the survival of microorganisms and plants in arid environments.

Future research should focus on the functional expression of the *acdS* gene and its impact on plant–microbe interactions under different environmental conditions. Additionally, exploring the horizontal transfer of the gene across different habitats could provide relevant insights into understanding the evolutionary mechanisms that enable bacteria to adapt and thrive in extreme environments.

## Figures and Tables

**Table 1:** Geographic distribution of study sites.

Location		Latitude/Longitude
El Cobre	C1	−24.2966 S/−70.4920 W
	C2	−24.3022 S/−70.4948 W
	C3	−24.3048 S/−70.5058 W
Quebrada Botija	B1	−24.5007 S/−70.5329 W
	B2	−24.5017 S/−70.5325 W
	B3	−24.5022 S/−70.5330 W
Quebrada Izcuña	I1	−24.4283 S/−70.5302 W
	I2	−24.4278 S/−70.5303 W
	I3	−24.4279 S/−70.5298 W

**Table 2:** Metagenomic assembly analysis for different study samples.

Samples	Number of Contigs	N50	L50
B1	118,985 contigs greater than 1000 bp	1279	70,268
B2	114,205 contigs greater than 1000 bp	1250	70,654
B3	119,063 contigs greater than 1000 bp	1230	75,425
C1	92,533 contigs greater than 1000 bp	1100	70,565
C2	55,944 contigs greater than 1000 bp	876	74,112
C3	60,780 contigs greater than 1000 bp	966	63,022
I1	112,580 contigs greater than 1000 bp	1202	80,825
I2	118,204 contigs greater than 1000 bp	1092	93,283
I3	61,411 contigs greater than 1000 bp	991	59,346

**Table 3:** Number of plasmids larger than 800 bp identified in different samples.

Samples	Number of Plasmids >800
C1	2779
C2	2558
C3	2502
B1	2788
B2	2832
B3	2832
I1	4600
I2	6121
I3	2636

**Table 4:** Identification of the *acdS* gene in plasmids.

Sample	% Identity	Coverage	Plasmid ID
C2	64%	240 aa	K141_255461
I1	76%	204 aa	K141_550940
I1	76%	316 aa	K141_772727
I2	60%	311 aa	K141_684709
I3	63%	245 aa	K141_341888

## Data Availability

The original contributions presented in this study are included in the article/Supplementary Materials. Further inquiries can be directed to the corresponding authors.

## Supplementary Materials

The following supporting information can be downloaded at: https://www.mdpi.com/article/10.3390/microorganisms13071547/s1, Figure S1: Taxonomic abundance of the microbial community at the different study sites.; Table S1: PERMANOVA analysis to support differences in beta diversity.; Table S2: PERMANOVA analysis using Location as a factor.
