# Supplementary material for: The Abundance and Distribution of the acdS Gene in Microbial Communities from the Rhizosphere of Copiapoa solaris, a Native Cactus in the Arid Coastal Region of Antofagasta, Chile
Source: Microorganisms. 2025 Jul 1;13(7):1547. doi: 10.3390/microorganisms13071547 (PMC12300068; doi:10.3390/microorganisms13071547)
Supplement: Supplementary file 1 [file microorganisms-13-01547-s001.zip › microorganisms-3649802-supplementary.pdf]

Appendix A (Supplementary Data)

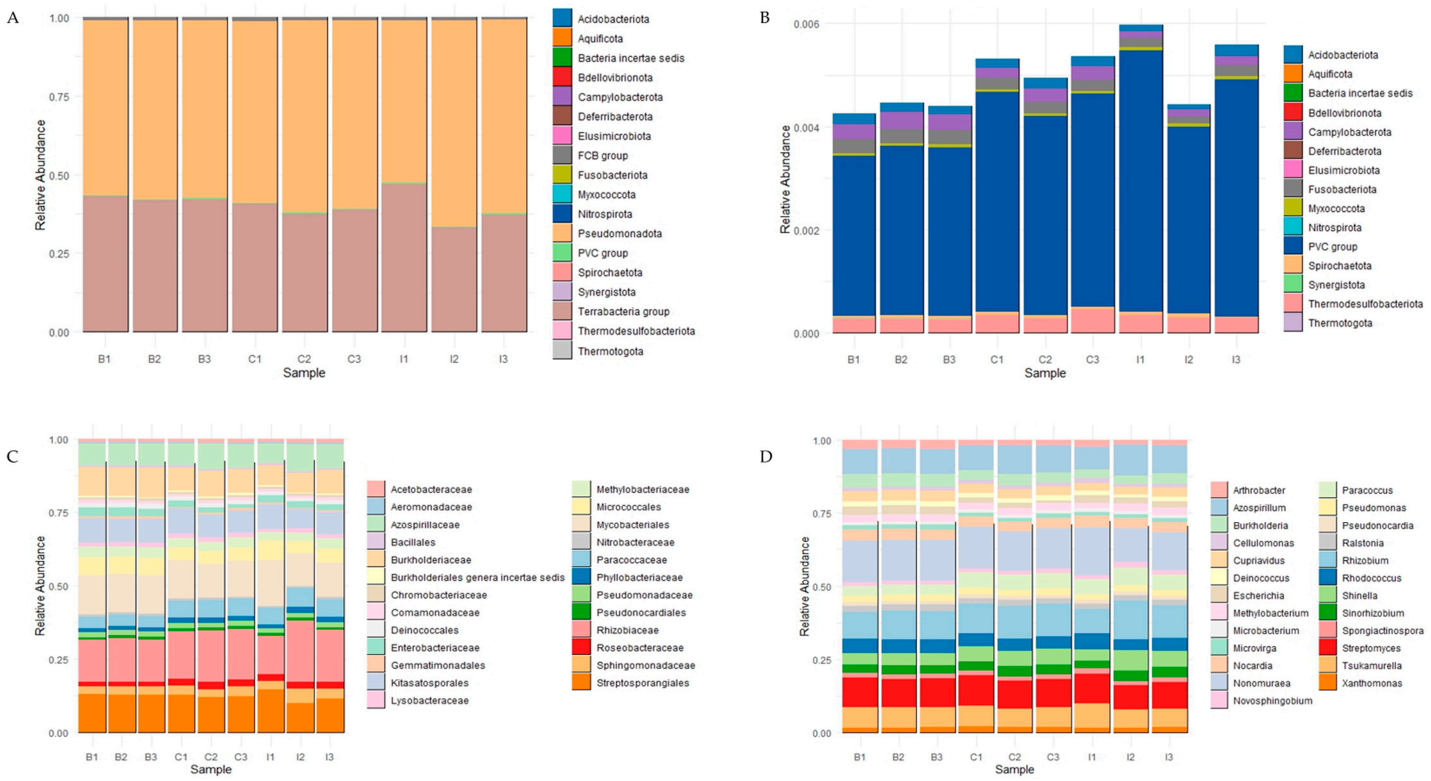

Figure S1: Taxonomic abundance of the microbial community at the different study sites, reflecting differences in composition according to the location of the three study sites: Quebrada Botija (B1, B2, B3), El Cobre (C1, C2, C3), and Quebrada Izcuña (I1, I2, I3). In A) the relative abundance at the level of the most abundant phyla is observed, where the predominance of *Terrabacteria* and *Pseudomonadata* is observed in all samples. In B) the relative abundance of the less abundant phyla (less than 1%) is observed, where the presence of *Pseudomonadata* in I1 and I3 stands out, while other phyla such as *Acidobacteriota* and *Fusobacteriota* are present in lesser proportions. In C) the relative abundance of the 25 most abundant phyla at the family level is observed, and a heterogeneous distribution is observed where the *Burkholderiaceae* family seems to be one of the most representative in the samples. In D) the relative abundance of the 25 most abundant phyla at the genus level is observed, where a similar distribution is observed between the samples, with some variations in the relative abundance of some phyla.

Table S1: PERMANOVA analysis to support differences in beta diversity.

	Df	SumOfSqs	R2	F	Pr (>F)
**Site**	2	0.02115	0.38128	1.8487	0.126
**Residual**	6	0.03432	0.61872		
**Total**	8	0.05547	1.00000		

Table S2: PERMANOVA analysis using location as a factor.

	Df	SumOfSqs	R2	F	Pr (>F)
**Location**	2	17.6903	0.73709	8.4109	0.005**
**Residual**	6	6.3097	0.26291		
**Total**	8	24.0000	1.00000		

Signif. Codes: 0, ´*`; 0.001, ´**`; 0.01, ´*`; 0.5, ´.`; 0.1, ´ ` 1.**
